# Process evaluation of the Invictus Pathways Program

**DOI:** 10.1371/journal.pone.0293756

**Published:** 2023-11-27

**Authors:** Dannielle Post, Amy Baker, Steven Milanese, Suzana Freegard, Gaynor Parfitt

**Affiliations:** 1 Alliance for Research in Exercise, Nutrition, and Activity (ARENA), Allied Health and Human Performance, University of South Australia, Adelaide, South Australia, Australia; 2 Allied Health and Human Performance, University of South Australia, Adelaide, South Australia, Australia; 3 International Centre for Allied Health Evidence (ICAHE), Allied Health and Human Performance, University of South Australia, Adelaide, South Australia, Australia; UNICAMP, University of Campinas, BRAZIL

## Abstract

**Introduction:**

UniSA’s Invictus Pathways Program (IPP), a service program, was originally developed to assist veterans to train for and participate in the Invictus Games. More recently, the scope of the IPP has widened to support and improve wellbeing and facilitate post traumatic growth and recovery among participants who are living with physical and mental health injuries and conditions. This paper describes the components of the IPP and reports its process evaluation.

**Methods:**

Underpinned by a pragmatic approach, data related to participant and student involvement in the IPP, the number of participant training sessions, session attendance, program activities and events, and program fidelity were compiled from process documentation that had been collected between 2017 and 2020, inclusive. Following ethics approval, semi-structured interviews were conducted with participants of the IPP, members of their family support network, and university staff to understand the operations of the IPP and satisfaction with the program.

**Findings:**

There was high fidelity for the student-led exercise training aspects of IPP; however, data collection relevant to participants’ psychological outcomes, and non-training IPP events and activities did not always occur as intended. Between 2017 and 2020, 53 veterans had participated in or were still participating in the IPP, and 63 allied health students had completed placements as student trainers. Fifty-three individual training sessions were delivered in 2017, increasing to 1,024 in 2020. Seventy-one interviews were completed with key IPP stakeholders. The qualitative analysis identified four higher order themes: Implementation and fidelity of the IPP, Satisfaction with the IPP, Areas of the IPP requiring improvement and suggestions for change, and Sustainability of the IPP. Satisfaction was generally high for the IPP, although there were factors that negatively impacted the experience for some participants and their family support network. Suggestions for improvement to program components and delivery aspects were made during the interviews, and the precariousness of IPP funding and sustainability was raised as an ongoing concern.

**Conclusion:**

This process evaluation has demonstrated that the physical activity training components of the IPP were delivered with high levels of fidelity, and that satisfaction with the IPP is mostly high, although there are areas that could be improved. There is a need for a more structured approach to the ongoing evaluation of the IPP. This includes ensuring that program staff have a shared understanding of the purpose of evaluation activities and that these activities occur as intended. Beyond this is the need to secure funding to support the sustainability of the IPP, so that it can continue to contribute to the wellbeing of veterans living with physical and mental health conditions, and their families.

## Introduction

The impact of military service is increasingly acknowledged as a key determinant of physical and psychological wellbeing for veterans and subsequently, their families [1–5]. Beyond the obvious health impacts, such as physical impairment or mental ill health, some veterans struggle to reintegrate into civilian society when they leave service [2,6]. This can lead to disengagement and isolation from their community, which can be further detrimental to physical and psychological health.

Strategies designed to support veterans and their families are important to ensure a strong and cohesive family unit that is physically active, socially engaged, and physically and psychologically healthy [4]. One approach to supporting veterans and by extension, their families, to achieve this is through engagement and participation in community-based adaptive sport and competitive sporting opportunities, such as the Invictus Games and the Warrior Games. Involvement in these adaptive activities has been shown to impact positively by increasing the physical activity of participating veterans and supporting their reintegration and rehabilitation [6–8].

To facilitate the involvement of current and former serving personnel in such opportunities, the Invictus Pathways Program (IPP) was developed, motivated by the spirit of the Invictus Games, to mobilise the benefits of sport to aid physical, psychological, and social wellbeing. As previously described [9], the IPP was developed in 2017 through a collaboration between the University of South Australia (UniSA) and The Road Home (now Military and Emergency Services Health Australia: MESHA). The purpose of this paper is to describe the IPP and to report the process evaluation of the IPP.

### The Invictus Pathways Program (IPP)

The IPP is a service program, delivered by UniSA Allied Health and Human Performance. It is intended to support and improve wellbeing and facilitate post traumatic growth among participants, who are living with physical and mental health injuries and conditions, using the power of exercise and adaptive sport. The IPP is comprised of two distinct but complementary programs: an Exercise and Performance Program (EaPP) and a Community Adaptive Sport Program (CASP).

Between 2017 and 2020, the EaPP was delivered to participating former and current Australian Defence Force (ADF) service personnel by third and fourth year UniSA allied health students, who are supervised in their placement within the IPP by appropriately qualified university staff. Embedded within UniSA’s curriculum, the IPP is intended to provide valuable interdisciplinary learning experiences for students across several disciplines, including physiotherapy, podiatry, exercise physiology, exercise science, and since 2020, occupational therapy. Students are recruited into the IPP Student Placement Program to complete a mandatory 120-hours of clinical placement over a period of 12 months. From a student perspective, engagement in the IPP provides a unique learning opportunity with a population of participants who have varied life experiences, as well as ongoing physical and psychological injuries. For participants, the EaPP provides opportunities to engage in physical activity, set and achieve performance and competitive goals, and access to allied health support, with a long-term aim of supporting community reintegration.

### The Exercise and Performance Program (EaPP)

Participants who enrol in the EaPP do so for a period of two years. At baseline, participants undergo screening assessment in UniSA’s allied health clinics (physiotherapy, podiatry, and exercise physiology), and complete background information (see measures within IPP). Ongoing management of conditions through the allied health clinics is provided as required for the duration of participants’ involvement in IPP.

Following these initial assessments, participants typically undertake a high-performance testing session, performed by an Accredited Exercise Scientist (AES) member of IPP staff, with support from AES or Accredited Exercise Physiology (AEP) students. At this stage, participants are asked about their functional aims, as well as the sports, events, and goals they have for participation and selection in sporting events. Based on this information, coupled with the outcomes of the allied health clinic assessments, a testing protocol is developed for the participant. This testing protocol may consist of maximal or submaximal testing, as well as assessment of maximal oxygen uptake (VO2max), blood lactate, peak power (sprint or sustained), body composition, muscular strength, or isokinetic dynamometry. Participants receive a detailed report that outlines their performance, how they compare to various population normative values, and where possible, relative HR or work rate-based training zones. It is intended that participants repeat this testing protocol every three months as a means of assessing the impact of their participation in IPP.

Participants are then assigned a student trainer, who works with that participant for the 12-month placement duration. Participants are assigned a second student for the duration of their second year in IPP. Where possible, students are matched to participants with respect to areas of sport or physical activity interest. Students deliver one-on-one exercise training to the participant at UniSA’s gymnasium facilities, with the regularity of session delivery dependent on the needs of the participant and the student’s availability. The student trainer, supervised by an AES/AEP member of the IPP staff, develops an individualised training program for the participant. This training program is reviewed every six weeks by the student trainer and IPP staff, with progressions and regressions, and the addition of new exercises as required. The student trainer then schedules regular training sessions with the participant, which are supervised by AES staff members of the university.

### The Community and Adaptive Sport Program (CASP)

The CASP is a suite of activities and adaptive sport opportunities offered in partnership with local community organisations. Some activities are in a ‘come-and-try’ format, whereas others are ongoing. The activities offered vary dependent on availability and include archery, cycling, kayaking, sailing, wheelchair sports, gym access, swimming, and rock climbing. The CASP approach is intended to facilitate the development of new knowledge and skills in a safe and social environment and support ongoing community engagement for veterans and their families, beyond their involvement in the IPP. CASP activities were coordinated by a Veteran Liaison Officer with lived experience and extensive engagement with veterans and community organisations.

The theory of change of the IPP is that engagement with student trainers, engagement with fellow participants with shared service experiences, participation in physical activity, and achievement of program-related goals, will result in improvements in physical and psychological wellbeing outcomes for participants. Through this process evaluation we sought to assess participant engagement and identify positive and negative influences on the implementation of IPP; and understand experiences and perspectives of those involved, as a means of examining the operation of the IPP and refining the program as necessary.

### Data collection and measures within the IPP

Survey data collection to assess the impact of participation in the IPP is intended to occur every six months, with baseline data collected as soon as possible following each participant’s enrolment in the IPP. The surveys used in the IPP, and their psychometric properties, have been reported previously [9]; briefly, these surveys collect information related to quality of life (WHOQOL-BREF [10] and VR-36 [11]), substance use (AUDIT [12]), mental health (Kessler-10 [13]), and post-traumatic stress (PCL) [14] and post-traumatic growth (PTGI) [15]. Physical performance data are intended to be collected at baseline and as part of three-monthly high performance testing review sessions. Overall program outcomes associated with these measures will be reported in a separate paper. In addition, a generic evaluation survey was developed for come-and-try activities offered by IPP, assessing engagement and satisfaction.

## Methodology

The IPP is what would be considered a complex program, with its multiple components and avenues of intervention. Involvement in the student exercise training, health care received from various allied health services, attendance at come-and-try events, opportunities to participate in CASP activities, selection or non-selection in the Invictus or Warrior Games teams, and engagement with other IPP participants all have the potential to impact the health and wellbeing of participants, yet not all participants will engage in every component or necessarily benefit from it if they do engage. Others may receive services external to the IPP, dependent on whether they are still serving. Consideration of these factors and the ‘real-world’ context in which the IPP operates is important in understanding what did or did not work in its implementation and delivery [16,17]. Further, exploring satisfaction with the program from the perspective of those it serves enables identification of components that require refinement or addition. Process evaluation is a necessary and important component of any evaluation; if we do not monitor or know if program components are being delivered or engaged with as intended, we cannot confidently attribute outcomes to specific components of the program, nor can we understand if the theory or mechanism of change worked as intended. It became apparent during data collection with IPP participants, undertaken as part of a PhD project to evaluate longitudinal participation in the IPP, that some participants had concerns about aspects of the program, its processes, or were not receiving the services they expected to. Furthermore, aspects of the evaluation framework (established during the development of the IPP) were not implemented nor adhered to as intended. It was because of this that a pragmatic approach to the process evaluation was chosen. We did not seek to prove or test a specific theory through this process evaluation but instead understand whether the theory or mechanism of change underpinning the IPP was working. The intention was to use the most appropriate methods available to us to address the purpose of the process evaluation, specifically, to identify whether the IPP was implemented as intended, to understand and describe satisfaction with the IPP; and to identify areas and components of the IPP perceived to be working well or which need attention and improvement.

## Methods

To undertake this process evaluation, data related to participant and student involvement in the IPP, the number of participant training sessions, session attendance, program activities and events, and program fidelity were compiled from process documentation that had been collected between 2017 and 2020, inclusive. Quantitative data were specific to the process evaluation of the IPP and did not include information that would enable the authors to identify program participants. Information related to participant satisfaction with the IPP and recommendations for program improvement and refinement was collated from semi-structured interviews undertaken with key IPP stakeholders, including IPP participants, members of the participants’ family support network, IPP staff, and members of the university’s staff and executive management team with vested interest in the IPP. Participants and members of the family support network were intended to be interviewed on more than one occasion, at approximately 12-month intervals; however, this was dependent on when the participant enrolled in the IPP. For example, participants who enrolled in 2020 would only complete one interview. In addition to assessing aspects related to process evaluation, interviews at multiple timepoints were intended to enable assessment of the impact of the IPP on physical and psychological factors over time, as well as to explore concepts related to participation in competitive games, such as Invictus Games and Warrior Games. Findings related to these aspects will be reported in a separate paper. Interviews with IPP and university staff occurred at one timepoint, with the purpose of the interviews to understand whether the IPP was being implemented as intended and the perceptions of staff and university management about what the IPP needed to be sustainable. Students undertaking placements in the IPP between 2017 and 2020 were not interviewed as part of this process evaluation.

### Ethical considerations

Ethical considerations relevant to this project have been reported elsewhere [9]. Briefly, due to the program being delivered as a service program, ethical approval was not required to deliver the program to veterans or collect data related to the day-to-day operation of the program itself, for example, appointment scheduling and attendance. As such, data collected in 2017 and early 2018 and included in the aggregated data reporting program delivery factors was done so prior to ethics approval for the collection of physical and psychological data. To enable the collection and analysis of data specific to the physical and psychological wellbeing of participating veterans, and to extend evaluation to include the perspectives of their family support network, ethical approval was required. This approval was sought from and provided by the Australian Departments of Defence and Veterans Affairs Human Research Ethics Committee (DDVA HREC, approved 8^th^ June 2018). Subsequent to receipt of this approval, institutional approval was provided by UniSA’s Human Research Ethics Committee (16^th^ June 2018). No program participants, members of their family support network or university staff were recruited to the interview components of this evaluation until ethics approval was received from both ethics committees.

Participants were required to provide informed written consent to be involved in the evaluation research. Prior to each interview, the aims of the research were explained to interview participants who were provided with the Participant Information and Consent Form (PICF). Participants who were current-serving members of the Australian Defence Force (ADF) received a copy of the PICF and a copy of the Guidelines for Volunteers to keep, as per the DDVA HREC’s requirements. To minimise the potential for discomfort to the IPP participants specifically, their interviews were conducted next to the university’s health service, so that immediate access to a General Practitioner could be provided in the event that a participant required additional support. Information about mental wellbeing support services was provided to everyone who participated in the interviews.

Interviews with members of the participants’ family support network were undertaken at the university campus or in cases where in-person interviews could not occur, via Zoom. Interviews with IPP staff and members of the university’s staff and executive management were also undertaken on campus. In 2020, social distancing and work-from-home requirements were implemented due to COVID-19 and following approval to amend the protocol, all interviews were performed over Zoom during this time. Audio recordings of interviews were stored on the university’s secure server and subsequently transcribed by the same member of the research team who undertook the interviews (SF). This researcher had sustained engagement with interviewees over the three-year data collection period and through this engagement had a good understanding of participants’ personalities and how best to engage with them during interviews, to elicit relevant information. Interview transcripts and other electronic data relevant to this process evaluation were stored on the university’s secure network and accessible only by members of the research team. The interview guides are provided in S1 File.

### Data analysis

Process evaluation data were collated into data management files and analysed to provide a descriptive overview of participation in the IPP and fidelity of the IPP. These data are reported as counts and percentages. Interview data were analysed using thematic analysis [18]. Following transcription, three random interviews were checked for transcription accuracy by a second researcher (AB). Throughout the data collection and data analysis process, peer debriefing was regularly undertaken within the research team as a means of exploring ideas and concepts but also to ensure that the preconceptions and previous experiences of the research team with this population were appropriately considered in the interpretation of data and the development of themes. This approach was intended to increase the rigour and trustworthiness of the analysis. It was intended that concepts identified would relate to program-specific and organisational operations, as well as participant satisfaction with IPP, with coding and themes developed using a mixture of deductive and inductive approaches. Interviews were initially coded by one researcher (DP) with first-round themes and their sub-themes collated. This researcher has a background in evaluation and assessment of psychological and physical wellbeing and had been a member of IPP’s Executive Committee since 2020; however, had no involvement in the day-to-day running or activities of the IPP. A second researcher (AB), also independent of the day-to-day operations of the IPP but with a long involvement in research investigating mental health wellbeing of this population, reviewed the data, codes and initial themes, refining as per her interpretation of the meaning of the data. Subsequent to this, themes were discussed and further refined (see S2 File, for an example of this process).

## Findings

### Participation in IPP

Between 2017 and 2020, 53 veterans had participated in or were still participating in the IPP. During this same period, 63 students completed placements as student trainers in the IPP; 38 of these students were completing a degree in Exercise and Sport Science, 25 students were completing a degree in Clinical Exercise Physiology. A yearly breakdown of participant involvement and student placement numbers is provided in Table 1.

**Table 1:** IPP participation and student placements, 2017–2020.

Year	Number of participants/year	Number of students/year
2017	12	8
2018	18	9
2019	36	19
2020	35	27

Participants’ attendance at student-delivered allied health appointments fluctuated between 46 percent for Podiatry in 2017 and 96 percent for Physiotherapy in 2017 and 2019 (Table 2). Seventy-seven percent of high-performance testing sessions (n = 26) were attended by participants across the four-year reporting period.

**Table 2:** Participant attendance at allied health appointments, 2017–2020.

	2017	2018	2019	2020
Attended/Booked (%)				
Exercise Physiology	17/22 (77.3)	45/56 (80.4)	61/102 (59.8)	35/43 (81.4)
Physiotherapy	22/23 (95.7)	89/94 (94.7)	136/142 (95.7)	75/84 (89.3)
Podiatry	6/13 (46.2)	14/15 (93.3)	31/50 (62.0)	35/44 (79.5)
Occupational Therapy	Not offered	Not offered	Not offered	32/37 (86.5)

In the first year of the IPP implementation, 53 student trainer-led sessions were booked, with 100 percent attendance. In 2020, the number of student trainer-led sessions booked increased to 1,081, with 95 percent of these sessions attended by participants (Table 3). Reasons for non-attendance were not documented. Surveys were distributed in 2018 to all 53 veterans who participated over the three-year period, of which 37 (69.8%) were completed and returned. Follow-up surveys were sent in 2019 and 2020, with response rates below 50% for both years.

**Table 3:** Student trainer-led training sessions with IPP participants, 2017–2020.

Year	Training sessions booked	Training sessions attended (%)
2017	53	53 (100.0)
2018	529	466 (88.1)
2019	1080	872 (80.7)
2020	1081	1024 (94.7)

In addition to the EaPP, 56 events and activities were delivered by the IPP team in 2020; no events or activities were documented prior to 2020. These events covered eight different types of activity, including cycling, indoor rowing, sailing, and wheelchair basketball. Attendance data were collected for all events, with a total of 213 attendees (Table 4). Data relevant to participant satisfaction with these events were not collected at the time of the event, despite the availability of an evaluation survey created for the purpose of collecting this information.

**Table 4:** Events and activities delivered and attendance 2020.

Event or activity	Number of times held	Total attendees
Cycling–coffee ride	10	51
Cycling–come and try	4	30
Cycling–hills ride	7	20
Indoor rowing	1	11
Sailing	1	5
Sitting volleyball	5	12
Swimming	25	71
Wheelchair basketball	3	13

### Qualitative findings

Seventy-one interviews were completed between August 2018 and January 2021. Forty-one interviews were completed with IPP participants, twenty-two with members of the participants’ family support network (FSN), three interviews with members of IPP staff, and five interviews with members of university staff and executive management.

Six IPP participants completed three interviews, seven IPP participants completed two interviews, and nine IPP participants completed one interview. One member of the FSN completed three interviews, eight members of the FSN completed two interviews, and three members of the FSN completed one interview. As previously indicated, staff of IPP and the university completed one interview.

Interviews ranged between nine minutes and 93 minutes, with an average duration of 26 minutes. While the brevity of some of the IPP participants’ interviews may be of concern, it reflects the nature of the IPP participants, many of whom were living with mental health conditions. The interviewer used their professional judgement and knowledge from their previous involvement with the participants to gauge when participants were no longer engaging in the interview process and as such, when it was appropriate to wind-up the interview. Despite this brevity, these participants provided key information and insight to their IPP experience, warranting their inclusion in the analysis. Information received from IPP participants and members of their FSN relating to areas of the IPP that were perceived to require immediate attention was relayed to IPP management directly after the interview, with the intention that strategies would be put in place to address the issues.

The main themes identified, and their sub-themes (Table 5), reflect concepts related to the Implementation and fidelity of the IPP, Satisfaction with the IPP, Areas of the IPP requiring improvement and suggestions for change, and Sustainability of the IPP. Quotations are provided to support the identified themes and their sub-themes, but these quotations are not exhaustive of the information provided relevant to each theme. To avoid identifying IPP and university staff who participated we have elected not to report information about the specific roles of the interviewees.

**Table 5:** Themes and sub-themes.

Theme	Sub-themes (as applicable)
Implementation and fidelity of the IPP	Impact of COVID-19 on implementation and fidelity of the IPP Impact of COVID-19 for IPP participants Barriers to engagement in the program and the impact on implementation fidelity
Satisfaction with the IPP	Program management, facilities, services, and personnel FSN perspectives: re-engaging participants in social and physical activities Dissatisfaction, disappointment, and negative perceptions
Areas of the IPP requiring improvement and suggestions for change	Program communication and scheduling Person-centred approaches, incorporating mental health awareness Transition pathway from IPP General suggestions for additions to the IPP to improve the experience Opportunities for peer connection and social engagement Family engagement and support
Sustainability of the IPP	Benefit versus expenditure Ongoing evaluation Online approaches to expanding reach and engagement

### Implementation and fidelity of the IPP

During the initial iteration of the IPP, there were multiple influences on the implementation fidelity of its components and activities, and subsequently, the capacity to deliver them with fidelity. These influences included staff changes in key roles in the IPP, which contributed to communication gaps and a lack of clarity around who was responsible for different IPP processes and what those processes were.

Interviewees involved in the delivery of allied health services within the IPP reported some difficulties in attempting to integrate the allied health disciplines to provide a multidisciplinary approach, there were perceptions of communication issues within and between disciplines, as well as concerns for academics in making sure that the involvement of their students in allied health service delivery aligned with teaching and learning requirements of their specific degrees. Each of these aspects contributed to delayed implementation, and in some case cases, ongoing impact on the fidelity of a truly multidisciplinary approach to service delivery within IPP.

I think there is some tension between disciplines around whose role is what, but that’s something we’ve identified and will continue to work on, is how best to work with each other and support each other (Staff 3, 2020)

I think the student placements not matching up across the disciplines, that’s a barrier. So, we can’t provide a really complete multi-disciplinary program for a person, because the students will have different timetables, they have different learning objectives…we’re trying to match up educational objectives with program objectives and they’re not always the same thing. (Staff 8, 2021)

Participants noted the disconnect between the length of time they had with their student trainers compared to the short-duration of other student placements and therefore a perceived lack of consistency in disciplines such as Physiotherapy, referring to lapses in communication between these services. This further reflects the misalignment of student placements across disciplines, and the potential for impact on participant outcomes as a result.

I know it was hard for my trainers, because they were having to chase down different physios all the time. So, whereas my trainers, I’ve had them for the whole year, whereas the physios were coming in I think it was every five or six weeks they had new physios…at one point last year they were actually able to sit down together. But then other times it was having to go sort of up through each of their chains to be able to communicate, to then be filtered back down…so there was a little bit lost in translation I think, with that process. (P10, interview 3, 2020)

Service delivery did not always occur as intended, with indications that fidelity of the associated processes was not met. IPP Participants reported communication issues related to follow-up appointments in more than one discipline, which resulted in those services not being received.

I had a physio assessment when I first joined the program, but I’ve had no communication since. Podiatry, there was a mix-up with my appointment, and they were meant to rebook me, but they never contacted me. (P18, interview 1, 2018)

The allied health services were perceived to be of value to participants who indicated that ‘just knowing they were available’ (P5, interview 2, 2019) was helpful; however, it was highlighted that there was potential for underuse of these services in cases where serving members of the ADF were required to use an ADF-provided service and other participants did not need to use the services. Concerns were raised about the equity of service access; while all participants were eligible for a physiotherapy screening appointment, only participants in the High-Performance arm of IPP were eligible for ongoing access at no charge. In contrast, members of the CASP arm of IPP who needed ongoing physiotherapy support were required to pay for it. Such factors also impact program fidelity and without a proper understanding of the aspects related to service use, the value of the allied health services to the IPP may be misinterpreted.

Fidelity of activity implementation and delivery were also reported to be impacted. Staff perceived that there was a lack of structure in the manner in which the IPP-associated activities were implemented, which led to inconsistency in their delivery and an apparent lack of communication to Participants about their availability. Staff identified the need for better and more consistent structuring of activities, rather than some of the ‘ad-hoc’ approaches that had occurred.

We need to be able to consistently offer activities…[for example], this [activity] happens every two weeks and there’s a staff member rostered on to do it, so that people are not having to sit around waiting on the Facebook group to see if something is actually going on. There really needs to be a schedule for the events. They can just be like, “It’s Sunday. I know this is on. I can show up if I want to.” I think some improvements need to be made there. (Staff 1, 2020)

Barriers to engagement in the program and the impact on implementation fidelity

Potential barriers to engaging veterans in the program were raised, including misconceptions associated with the name of the program and what the program was about. Issues such as these have the potential to reduce the appeal of the program to the target population and lead to poor implementation and delivery fidelity. Staff spoke about this and how the IPP could be better represented by staff.

I think because it’s called the Invictus Pathways it’s actually limiting it, to be honest. Because it’s making it sort of a bit elitist in that group. We’ve got so many veterans here that could benefit from the program that don’t want to be at that level. (FSN P26, interview 2, 2019)

Some veterans are apprehensive, because they don’t want to compete. They see Invictus–but Invictus was just a ripple effect. It just means ‘unconquered’. So, and Pathways means you can go into different avenues within the program at any time or any point in your life. So, a fifty-year-old veteran who’s got a knee injury and a lower back injury, doesn’t want to compete, but wants to just out and feel some wellbeing…And we are gradually getting that message across. (Staff 4, 2020)

That’s probably one of the bigger improvements that we’re trying to look at is also the way that the staff project it [the program], to everyone…just the way we communicate things, I guess. (Staff 1, 2020)

### Impact of COVID-19 on implementation and fidelity of the IPP

In 2020, the COVID-19 pandemic had a major impact on the capacity of program management to deliver the IPP as was intended. While the impact was felt by participants, there was also the need to consider the impact on student trainers and their placement requirements, in addition to managing the day-to-day operations and activities offered by the IPP. All on-campus allied health clinics were closed, as was the gymnasium, and university staff worked from home for approximately four months. Staff members described the positive approach to the circumstances by students and staff, acknowledging their support of participants and shifting to an online delivery format in a short period of time. The circumstances brought with it opportunities for students to experience delivery of support in a telehealth format and presented options for consideration of approaches to expansion and future delivery of the IPP.

…with the initial shock of everything shutting down completely, then the great initiative of some of the staff within UniSA, and with the students, we created online Zoom platforms. (Staff 4, 2020)

From the student point of view, I think they did an absolutely fantastic job in delivering the services. We all went online within three days or whatever it was…I think there’s some learning that the students will take forward around telehealth, and remote service provision, to people who are not able to get into the city, for example…There’s no reason why students couldn’t provide this same level of support to those that live in rural or remote communities, around exercise and sport. (Staff 3, 2020)

### Impact of COVID-19 for IPP participants

Participants reported that their training initially came to a halt due to COVID-19. One participant acknowledged that while the Zoom sessions were ‘not the same as having your trainer there, breathing down your neck, it was something’ (P7, interview 3, 2020). Others acknowledged that the online approach enabled participants to continue their training, maintain their motivation and a sense of structure, and remain socially connected; however, this was not the case for all.

The participant aspect, I think they were more heavily impacted than the student group, mainly because most of our clients have mental health illness—or fluctuations in their mental health—that was really adversely affected by COVID full stop, as a lot of the population was. But also, then, we weren’t able to do the one-to-one services that we wanted to provide, and we also weren’t able to provide a lot of the community-based supports. You know, things like a coffee ride on a Friday morning, or a breakfast ride on a Sunday morning, where people would come, and yes, they would do their physical activity, but perhaps the more important aspect was the social aspect, and they were catching up with mates and having a coffee and a laugh and things like that. That’s what participants really missed. (Staff 3, 2020)

My trainers have saved me by–I’ve been training with one of my trainers online via Zoom at least twice a week and doing the group sessions whenever I could. I feel like if it wasn’t for those Zoom sessions, I would have completely lost all motivation and gone back to where I started 18 months ago. So that has been an absolute godsend for me, because I committed to doing it so then I had to do it. Otherwise, I would have just done nothing. (P21, interview 2, 2020)

I know [IPP staff] were doing everything they could to reach out through social media, and through the links that we established…trying to get group classes through Zoom, trying to do social rides on Zwift and stuff like that. So I think the staff were doing everything they possibly can with the restrictions they had, and it really comes down to the individual and how much were they really willing to get off their backsides and participate in it. Unfortunately, I didn’t have that motivation. (P4, interview 3, 2020)

### Satisfaction with the IPP

**Program management, facilities, services, and personnel.** For the most part, participants reported that they were satisfied with the IPP and its components, including management of the program by the university, availability and access to allied health services and facilities, and the support given to participants across multiple levels of the program. This included acknowledgement of the support from student trainers, allied health clinic staff, IPP staff and the university team generally, and perceptions that the IPP offered a ‘safe’ environment for participants living with mental health issues.

There’s an investment from the students and, you know, particularly [EaPP staff member] as well, he has followed them all the way through. He’s been there. And, you know…it’s been the investment and the buy-in. I think for [participants] they can see, you know, that people actually care…that they do give a shit and it’s not just…we’re not just tokenistic and it’s, you know, we’re ticking a box that we’ve been involved with veterans, which is what quite a lot of organisations do. (FSN5, interview 1, 2018)

I’ve been through the military rehab, this seems to be the best program I’ve ever come across. And you’ve got the medical, you’ve got the professionals…You know, you’ve got the EP. You’ve got the likes of [IPP staff member]. You’ve got the physios. You’ve got the doctors. You’ve got everybody here. (P4, interview 1, 2018)

One of the big things for me, is a place I feel safe, I feel comfortable coming here, whereas I don’t like to go to other gyms, because I don’t like the noise, or too busy, the crowdedness and that sort of stuff. (P3, interview 2, 2019)

Participants also spoke about the beneficial aspects of the delivery of the IPP, for example one-to-one training, engagement with students, and the program being structured to change participants’ mindsets and not work from a deficit model.

…it’s more personalised here because you’re that one person that they’re seeing all the time and it’s more about you, you’re not just the client amongst 100 others that they’ll see. (P20, interview 1, 2019)

I feel just that training, that accountability training with them [students], that is—it’s honestly, priceless. It’s going to be sad when it’s my time to give that up, because then I have to—it’s all on me then. (P20, interview 2, 2020)

So, the Exercise Physiology department got me into something new, got me into [a] different mindset of it’s not what you can’t do, it’s what you can do. (P18, interview 1, 2018)

### FSN perspectives: Re-engaging participants in social and physical activities

Members of the participants’ family support network mostly perceived the IPP to be a ‘fantastic’ program, re-engaging participants in social and physical activities. One interviewee professed their thanks to the program for giving their participating family member ‘their life back’. In a follow-up interview, the same family member stated that they wished the program had been around earlier to support other family members in their transition from service to civilian life.

So when Invictus came his way and he joined the Program, everything changed and he could see the light at the end of the tunnel, you know. Then he realised he could do cycling. He could other sports and you could see the twinkle, twinkling sort of lights in his eyes. It’s like ‘yeah, I can do it!’ (FSN4, interview 1, 2018)

But all I can say [is] thank you UniSA and The Road Home program for giving my [family member] back his life. I tell everyone about it because I just think it’s amazing. (FSN7, interview 1, 2018)

Just please don’t stop it [the program]. Not only for my [family member], but for the other veterans out there…Please don’t stop it, because I wish there had of been something like that round when my [other family member] got out of Defence. I’m a mother, and I’m also a wife of a veteran that has suffered, so I can see the benefits hugely that it’s done for our son, but I do wish it was around a lot earlier. (FSN7, interview 2, 2019)

### Dissatisfaction, disappointment, and negative perceptions

A negative influence on satisfaction with the IPP was related to a factor that was outside of the control of the management of the IPP: selection or non-selection for the Australian Team for the Invictus Games. While external to the activities of the IPP, this factor reflects the multilevel influences on the mechanisms of change within IPP and subsequently, program outcomes. The impact was reported by some interviewees to be significant, and it would be remiss not to address it here. Selection was the responsibility of the ADF; however, the involvement of IPP staff in supporting ADF training camps, and the perception that ‘there seems to be a lot of politics’ (P4, interview 2, 2019) or ‘favouritism’ in the selection process was raised by multiple participants and members of their family support networks as a point of concern and contention. For some, the personal impact of this process and non-selection in the national team resulted in mental health impacts that required hospitalisation or making the decision to remove themselves from some IPP activities or to leave the IPP entirely. It is important to note here that processes associated with Australian team selection have since been refined by the ADF, specifically, greater transparency around selection requirements. Further, the feedback from interviewees identified the need for the IPP to better support participants whose goal to achieve national team selection is not reached.

Some members of the family support network expressed anger and disappointment on behalf of their participating family member, based on the experiences of the family member regarding the previously mentioned issues with national team selection and perceived politics within the IPP itself. There were concerns that participants were ‘not followed-up’ or adequately supported by the IPP staff or by fellow participants when they missed team selection. These concepts were reiterated by some participants–and across multiple interviews–who reflected on wondering what they had done wrong, why they were no longer treated as ‘part of the team’ (P2, interview 1, 2018).

To me that is where the Invictus Pathway falls down. Granted, they gotta concentrate on the athletes that have got through and all the arrangements and all that stuff, but do not leave the other guys who have not got through. It’s like ’Okay, you’ve had your chance. We’re not interested in you anymore.’ This should be a continuous thing on with the rest of the guys in the Invictus Pathway to make them still feel they’re part of it. (FSN2, interview 1, 2018)

The need to better support participants who did not achieve their program-associated aims generally, not limited to selection for competitive events, or who seemed to not be engaging in IPP activities was also raised. A small number of participants and members of the family support network perceived that there was ‘no interest in you anymore’ at the program-level for participants who were not performing, or that some participants received preferential treatment and were ‘on pedestals’ (P3, interview 3, 2020) due to their achievements at competitive events. The mental health status of the participants as an influence on these perceptions was also articulated.

…I honestly feel like if you’re performing and you’re on the transition to get medals and make the whole program look great, then everybody is clapping you and on your side. But as soon as you’re not in that position anymore it’s like there’s no interest in you anymore. And whether that’s their intention or not, that’s sometimes how you feel. And that’s mental health, you know? (P3, interview 3, 2020)

…while it’s a fantastic program there tends to be a lot of focus and a lot of promotion on the medal winners and who’s winning. So those that are sort of struggling with the mental health aspect which is finding it hard to get onto the fitness train, I think they’re falling off a little bit by the wayside. (FSN3, interview 2, 2019)

The introduction of new approaches was influenced by what staff believed to be an ‘organic’ shift in IPP focus from one of high performance to a more holistic health and wellbeing approach, as a result of staff beginning to get an understanding of what participants needed.

We looked at so many of the participants and thought, ‘Winning a gold medal is good but that’s not necessarily going to be the thing that will keep them alive in the long term’. So, really, it’s been an organic thing that’s been driven by the goals of the participants and how we interpreted those goals within the program. (Staff 2, 2020)

A few participants described dissatisfaction associated with not achieving their goals, not receiving the services they expected to while participating in IPP, for example, the full extent of student training, not being contacted for follow-up by allied health disciplines, being provided with limited information about program phases or activities, and having negative interactions with IPP staff members or staff associated with IPP components.

I would say no, it hasn’t been a beneficial experience at all. I haven’t come away with any sort of lasting friendships, I haven’t come away with any of fitness achieved or anything like that, any sort of personal goals achieved or anything. (P17, interview 3, 2020)

The communication during the entire process was very poor and I was always being told, ’We’ll contact you. Don’t call us, we’ll call you,’ and I was waiting and waiting and waiting and nothing ever happened. Even down to everyday training routines and things like that, I kept on asking, ‘If I can’t come there, can you give me some worksheets or something I can work with and go to the local gym and at least train?’ but those things never happened so it’s just a bit of a shame they didn’t. (P2, interview 2, 2020)But more emails, more regular emails would have…especially at the beginning telling you especially things about okay, these are the sports you’re going out for, here’s the information on–or this is where you go to find the information on how the competition runs, and all that sort of thing. (P14, interview 1, 2019)

Yeah so I left the program…the relationship and the feeling from the [program component] was pretty poor. (P3, interview 3, 2020).

### Areas of the IPP identified as requiring improvement, and suggestions for change

This theme incorporates sub-themes that represent issues that interview participants experienced and, in their view, need to be addressed, as well as factors that interview participants believe will enhance and improve the general experience of involvement in IPP for participants.

### Program communication and scheduling

IPP participants, members of their family support network, and IPP and university staff all raised areas for improvement, with communication highlighted multiple times. This included timely communication to new participants and their family members about how IPP is structured, the scheduling of IPP activities, as well as communication with participants more generally, and the platforms used for communication. For one participant, the timing of the Zoom sessions during COVID lockdowns and the need to use technology was reported as being a barrier to involvement and could be considered an area for improvement. One participant and their family support network reported that they did not receive any contact from their trainers or offers to provide ‘at home’ workouts during COVID. (FSN PS43, interview 2, 2020).

What I don’t have right now is that appreciation of how the Invictus Pathways Program moves into the Invictus system. So, I would love a bit more knowledge about how it works so that I know what I’m working for. It seems a bit of a grey area for me at the moment. (P11, interview 1, 2019)

Probably the only thing that I find is that the program seems to—well, I’m not sure what part of the program he’s in at the moment, so we haven’t seen a chart that says, ’You’re here and this is where you’re going to in the long run,’ and all that. (FSN11, interview 1, 2019)

Yeah, I would have loved a lot more information, not just ‘go to Facebook’. Like I don’t use Facebook very well, I don’t use it much. I can’t use any social media at work, I can’t take my mobile phone into work, so I have to email, you have to email me. (P14, interview 1, 2019)

### Person-centred approaches, incorporating mental health awareness

The need for a person-centred approach and structuring processes to accommodate the fact that the majority of IPP participants are living with mental health issues was raised. This included the need to consider the likelihood of engagement in events by participants living with anxiety and how to best facilitate engagement for these participants.

We found that a lot of participants on their forms were ticking they were interested in all these things, but they never actually ended up there…they were given the information about it [an event] but they’re new, they don’t know anybody in the program, they weren’t really connected properly, so for them, especially with some of the anxiety issues and stuff like that that they have, it’s a big thing for them to just show up. (Staff 1, 2020)

Another participant, perceived that staff associated with a specific service provided within the IPP did not have a good understanding of the mental health of veterans. The participant’s experiences with the staff in this situation impacted their satisfaction with aspects of the IPP.

So, for the most part that’s been really positive here…but I think some parts of the [program]…I’m sure they just don’t understand our mental health, and maybe if they’re not, maybe they should be made aware of what our triggers are. I definitely felt like at some points in time, I felt like I wasn’t welcome anymore [at service]. (P3, interview 2, 2019)

Considerations about the mental health of participants extended to the previously noted concerns around communication, with suggestions to respond to program applications and inquiries promptly and to provide detailed information about program activities and the expectations of participants in the program. How an untimely response or lack of communication manifests for a participant living with anxiety is described by the following scenario, recounted by a participant:

When I first put my application in. I put in in, and there was no acknowledgement that you have even submitted it, and there is this whole uncertain–I was already uncertain about putting it in and wasn’t sure if I wanted to do it, and so it wasn’t until I got a call from UniSA that I was like, ‘Okay. This is all going ahead.’…but it is really nerve-wracking when you first put it in. You are like, ’Did anyone get that? Didn’t they get it? I didn’t even really want to do it anyway. Maybe I should just go back to sitting on the couch.’ (P21, interview 1, 2019)

Continuing with the importance of person-centred service provision, staff supported the inclusion of occupational therapy placement students in the IPP in 2020. These students receive some mental health training in their degrees, which is not provided to students in other allied health and exercise-specific disciplines. The value of these students and the potential of their role in supporting IPP participants was acknowledged by staff.

One of the reasons we brought the OT students in is because the OT students have mental health training…They’re getting all the information from the participant on where they’re at, and what their conditions and things like that, physical and mental, and then being able to decide what they need to help with that and if they need ongoing OT, or if they need other referrals and stuff. Whereas the other allied health disciplines just wouldn’t be able to do it. (Staff 1, 2020)

### Transition pathway from IPP

Participants spoke about the need for a clear and supported transition pathway out of IPP. This included the availability of pathways to other activities and community sports as well as information about who to contact for these opportunities. Participants also described feeling that they did not successfully transition from the IPP, that they were not ‘in a great place’ (P3, interview 3, 2020) and felt they had lost all their support. Comparisons were made to the lack of transition from the military and how the perceived lack of transition support from IPP was creating the same issues for participants.

There needs to be a way for people to transition out and to continue on. Whether that’s that you’re partnering with vocation[al] type of thing…maybe it’s work, maybe it’s getting themselves back into whatever it is, or maybe potentially partnering more with community organisations, community sports and stuff like that. (P22, interview 1, 2019)

We’re in this position in the first place because we joined the military and we got turned into something and there was no transition in getting out. So in some cases, they’re [the IPP] actually causing the same problem that we started with because you feel like all of a sudden, you’re not important and you’re not part of anything. (P3, interview 3, 2020)

The provision of appropriate transition support was noted by staff too, who discussed the need to consider the impact of removing one-to-one support for ongoing participation in physical activity, external to involvement in IPP, and to have a structured timeframe in place to help prepare participants for transition from IPP. A lack of foresight around the need for these processes during IPP development was acknowledged by IPP staff.

I think that’s probably something that—when we set up the program—we didn’t initially foresee, about what happens when you start to withdraw supporting services, and from an OT point of view and an allied health point of view, we’re very much about that, as how the participant can maintain that program once we’re not involved. (Staff 3, 2020)

…if you put a transition in place, which is what we’re doing with the new participants, saying, ’At 18 months we can start transitioning you.’…it actually gives them a really set structured timeframe goal, which is really important, I think, for people, for therapy. (Staff 7, 2021)

### General suggestions for additions to the IPP to improve the experience

Suggestions to improve the IPP experience covered vast areas, from suggestions about program staffing and program components, to suggestions about approaches to how students train participants. For example, some participants perceived that they needed to ‘fit-in’ with the students’ academic commitments, rather than sessions being scheduled to suit the veteran. For others, the university shut-down period and lack of access to students and facilities over the Christmas and New Year break was raised as a possible issue that could lead to participants ‘falling in a hole’ or losing motivation to train.

So I feel like maybe if the program didn’t have such–the same break then perhaps I wouldn’t of gone so far down. Because if I was still expected to show up for an active recovery session even once a week or what have you, then perhaps I would have not gone as far down as I did, or it might not have lasted as long. (P20, interview 1, 2019)

One participant voiced their surprise at not being able to provide feedback about their student trainer, suggesting that being able to do so would be valuable for the IPP staff and also for the ongoing development of the students themselves.

…I suppose one thing that surprised me was the, in a sense the lack of feedback from a participant’s point of view in regards to the students’ performance. So, if you’ve got say two or three trainers, you could potentially, as a participant, give a unique perspective on how you think they’re doing with the training regime to the people here who may have not had that oversight of them in the field. (P5, interview 1, 2018)

### Opportunities for peer connection and social engagement

Participants suggested that having more opportunities for IPP participants to connect with each other is important, and staff noted that IPP participants who have previously transitioned from the ADF could support participants who are in the process of transitioning from the ADF whilst involved in the IPP (Staff 1, 2020). This approach to engagement was further supported by other members of staff, who spoke about IPP participants wanting to act as mentors to new participants, to model their experience for others: ’We’ve done it. We’ve learnt it. We’ve lived it. We want to help others to do the same.’ (Staff 7, 2021)

…one of the things that I’ve probably noticed in the last few months is that a lot of the participants, if they’re current transitioning from the Defence Force, it’s a really stressful time for them and so having them involved in the program at that time is really beneficial, I think, for them…I think it’s actually really good for them to be engaged with the other participants, who may have gone through the same things and can give them some advice and things like that. (Staff 1, 2020)

Toward the end of 2020 and as COVID restrictions were removed, IPP management sought to re-engage the participants, beyond just their involvement in the training, but instead to provide opportunities for people to talk to each other and interact.

We really thought it was important to do some group things towards the end of this year, when we were allowed to, so that we could bring everybody together…We really wanted to choose an activity that was more of a social activity than like everybody going and riding bikes or something, where you can’t really talk to everybody, and we wanted a social kind of family event. We did a couple of those things towards the end of this year to really get the group back together. (Staff 1, 2020)

The inclusion of social events as a means of engagement and ongoing support and of conveying information was highlighted as being important to participants themselves.

And even if we had a social catch-up or whatever, because I know there’s a lot of people in the program, and a lot of us don’t know each other. If we bump into each other at the gym, or we share a sport, that might be the only time that we see each other. (P9, interview 2, 2020)

I’d like to see some social functions, like let’s all meet up and have a coffee and discuss—maybe somebody give a lecture or a presentation on how the whole program works…Right at the start, a bit of a social get together with the old hands welcoming the new guys and that be over a coffee or something. You can have a bit of a chat and ask all the dumb questions you’ve got. I think that might be a good thing. (P11, interview 2, 2020)

### Family engagement and support

The connection with family, the potential to impact family-unit wellbeing, and greater involvement of participants’ families in the activities of the IPP was raised as important by IPP participants, their family support network, and staff. Family members spoke about being part of ‘the journey’ and it being their journey also, experiencing the ‘highs and lows’ with the participants. (FSN4, interview 1, 2018)

I think as a family, I mean we are busy. But it would have been lovely if we could have been part of it a little bit more. (FSN5, interview 1, 2020)

…there’s a key gap in and around supporting those family, children and those that are supporting a veteran…So I think that’s a really clear opportunity for all service providers, but something that we also need to give some stronger consideration to, as well. (Staff 3, 2020)

Oh, probably we have become more close [due to participation in IPP]. And he needs the support and he knows that I’ve got his back…Sometimes he’s gone for a week or a couple of weeks, you know. But I know it’s doing good for his wellbeing, for our wellbeing, for our relationship. (FSN4, interview 1, 2018)

### Sustainability of the IPP

This theme included three sub-themes of ‘benefit versus expenditure’, ‘ongoing evaluation’ and ‘online approaches to expanding reach and engagement’ of the program.

### Benefit versus expenditure

The preliminary qualitative evidence for the benefit of participation in the IPP, the acknowledgement of the potential for long term impact of the IPP, as well as the support of the participants and members of their family support networks to keep the IPP going is countered by the expense to do so. This includes the costs associated with facility and equipment provision, staffing and managing the IPP, and supervising students. Further, there is the need to ensure that the program is meeting the core business of a university—educating its students.

From my perspective, the expenditure and the size of the program has been the biggest challenge. It’s a good challenge to have, particularly given that we need the students to get authentic placement experiences as part of their degrees. (Staff 2, 2020)

Obviously, I’m very passionate about this program and I’m really passionate to see that it continues to thrive…justified on the basis that it’s a good thing to do, provides a great service, but it’s providing a unique work experience for our students. There is no other university that is providing this type of experience on a structured basis for students in allied health. (Staff 6, 2020)

### Ongoing evaluation

Beyond this is ensuring that there is capacity to undertake ongoing refinement of the program, through adherence to an implementation and evaluation framework, to support the needs of the students and the needs of participants as the IPP continues to grow. The need to demonstrate the impact of the IPP to justify ongoing expense by the university and potentially engage external sponsorship was acknowledged by staff, as was the challenge in obtaining funding for this type of program and evaluation activities.

It comes back to financial support to do the evaluation. From a research point of view, we all know that funding is really hard to get, and funding that wants to show effectiveness of a community-based program here is really hard to obtain. (Staff 3, 2020)

The research costs money, and obviously we need funding to evaluate the program to—and that’s exactly the sort of thing that–the type of research that you’re doing, is looking at the impact this program has on individuals, and also on the students who are helping with the program. (Staff 6, 2020)

### Online approaches to expanded reach and engagement

Lessons learned during the COVID-19 pandemic, around using online technologies to support participants may be transferable to approaches to supporting participants who live rurally or remotely. This would not only support IPP sustainability, but also contribute to supporting veteran populations who are underserved or typically and incorrectly labelled as ‘hard-to-reach’.

There’s no reason why students couldn’t provide this same level of support to those that live in rural or remote communities, around exercise and sport. So I think from a student learning point of view there’s some key lessons that we can take forward, and that we’ll be looking to use when we think about expansion in the New Year, is maybe the big city sites are not the place that we need to expand to. (Staff 3, 2020)

## Discussion

The purpose of this process evaluation was to explore the implementation and delivery of the IPP between 2017 and 2020, with specific attention on concepts related to fidelity, satisfaction from the perspectives of key stakeholders, identification of aspects that require improvement, and program sustainability. Members of university staff perceived difficulties in integrating the allied health disciplines to provide a truly multidisciplinary service and this was reflected in the feedback of some participants, who thought there were communication issues between disciplines. Despite this, process data suggest that the fidelity of implementation and delivery of IPP EaPP-specific activities was high, as was attendance at allied health appointments, for the majority of disciplines, and attendance at exercise sessions with student trainers, despite interruptions associated with the COVID-19 pandemic toward the end of the evaluation reporting period.

The importance of process evaluation, the need for structured evaluation frameworks and appropriate implementation of and adherence to an evaluation framework is widely acknowledged. This is a unique program, with multiple intervention touchpoints, and avenues for impact. There is scarce recent literature reporting process evaluations of physical activity programs for veterans, with qualitative exploration of participants’ perceptions further limited. Where program attendance has been reported, it is generally high; however, the programs are 12 weeks duration and are not comparable to the two-year duration of IPP. The intention of the IPP is to not only support the physical and psychological wellbeing of veterans through engagement in physical activity but also to support the learning and development of allied health students through placement opportunities that enable them to work with real-life participants. Effectively managing the needs of the IPP’s participants in the context of the core business of the university and its operational environment is necessary for success of the program. The findings of this process evaluation will be useful for those trying to implement similar student-delivered programs in university environments and are also relevant to other programs that are intended for veteran populations.

Adherence to evaluation processes such as the collection and documentation of relevant project information, the regularity of survey distribution, and evaluation of one-off or come-and-try events did not occur as intended. This reflected a lack of understanding by some staff of the importance and reason for evaluation, both as a means of demonstrating the impact of the program for participants and in supporting ongoing funding of the program. Further contributing was a lack of clarity around responsibility for the role of survey distribution and follow-up, mostly as a result of staff changes. Low response rates to the second and third rounds of surveys may further reflect the lack of value of the evaluation held by staff, who may not have voiced the importance of survey completion to participants nor followed-up participants who received surveys. Beyond this is the potential influence of the research fatigue expressed by IPP Participants and reported anecdotally by members of IPP staff and family members of veterans in previous research [4]. While survey response rates of 50 percent and higher are considered good, a recent meta-analysis identified that average response rates to online surveys is 44.1 percent [19]. This suggests that the low response rates are not particular to this veteran population; however, it is necessary to identify approaches to better engage this population in the evaluation components of the IPP.

Participants and members of their family support network expressed satisfaction with the program and its components for the most part, in particular, access to the facilities of the university and its program partners, and the allied health clinics, as well as the support provided by student trainers and program staff. Positive impacts associated with social support from program staff and fellow participants have been demonstrated in the evaluation of a physical activity program for older veterans living with PTSD [20]. IPP staff described the ‘organic’ evolution of the program over this time and the shift from a high-performance focus to one that was intended to better incorporate the social and emotional wellbeing of participants, and provide opportunities for ongoing community engagement and integration, beyond participation in the IPP. Further distinction of the program from the Invictus Games and consideration of re-naming the IPP may alleviate the identified barriers to participation associated with the name and the connotation that involvement in the games is an expectation of participation in IPP or that the program is only for people who want to participate in the Invictus Games.

The involvement and commitment of the student trainers was highly valued by participants, and the strong working relationships forged between participants and their student trainers provided a sense of accountability and motivation for participants to engage in their training. These perceptions were reflected in the high proportion of training sessions attended. Participants appreciated the support of the IPP team and university staff generally, as well as the availability of allied health services, and the use of the facilities provided as part of the program. Participants also appreciated the opportunity to engage with other participants who had served or were serving in the ADF, and reported feeling a sense of belonging, being part of a team, and for some, being in a safe environment when they were involved in the program. These findings bode well for supporting the intended mechanism of change of the program; however, establishing psychological outcomes during this period using objective quantitative measures is not possible due to the low survey response rates. An understanding of how to better engage veterans in the evaluation of the IPP and to ensure the IPP meets the needs of the participants may occur through the creation of a stakeholder group that supports collaboration between IPP participants, their families, IPP staff, and evaluators [21].

Despite this general level of satisfaction, a number of factors were identified as needing improvement by interviewees, including process-specific and participant-specific components. These included communication at multiple levels of the program, in particular ensuring that relevant program information was made available to participants and that their queries and requests were acknowledged in a timely manner; structured processes for transition that build capacity and do not leave some transitioning participants experiencing the same negative feelings they associate with transition from Defence; and greater involvement of families in program activities and acknowledgement of the role families play in providing support for the veteran [22,23].

Although the IPP evolved to offer a more holistic approach to wellbeing, some participants still join the IPP with the intention of gaining selection in the Invictus Games team. Not having strategies in place to appropriately support participants who did not achieve selection was a major oversight in the implementation of IPP, particularly in light of the mental health status of the majority of participants. This was likely due to the small number of participants in the early iterations of the program and the fact that most who sought a place in the team were selected; however, it may also have just been perceived to be outside of the scope of the program or the responsibility of the ADF, who manage the Invictus Games team. The experiences of veterans who compete in representative teams may be similar to those of Olympic athletes who experience ‘post-games blues’ [24], and measures should be put in place to not only support veterans on their return but also prepare them appropriately for the aspects of competition that have the potential to further exacerbate existing mental health issues [25]. Further, it was perceived that participants who did not do as well as anticipated at the games were also not supported when they returned. Despite acknowledgement by IPP staff that broader support was required, this came too late for a small number of participants who chose to leave IPP.

The role IPP participants play in supporting students’ learning and the sense of ‘continuing to serve’ this brings has been described previously [9]. The opportunity to provide mentorship to others has been shown to be a reason for continued participation of veterans in physical activity [26] and formal strategies that enable IPP participants to do this for their fellow participants may contribute to an enhanced approach to support that has been identified by some participants and members of their family support network to be missing in the IPP.

Sustainability of the IPP requires these aspects be addressed and ensuring that those involved in the delivery of the program and its components have the knowledge, skills, and qualifications to work effectively with veterans living with mental health issues. This includes the provision of services and support that cater to the specific needs of veterans and their families. The trauma-informed approach [27] to the IPP is reflected in some findings, for example, with respect to the sense of safety felt, and the trust and collaboration established between participants and their student trainers. However, implementation of these principles needs to be re-visited in the IPP-associated services where participants reported feeling like they did not belong or were not welcome. Currently in Australia, principles related to military-friendly campuses are not as widely employed as they are in the United States and Canada, for example [28]. Extending concepts associated with student-centric military cultural competency training [29] across the university and its services may also contribute to an enhanced sense of belonging and support for IPP participants. For example, this would include increasing awareness of the issues IPP participants and their families may be living with.

The number of participants and student trainer placements almost tripled across the four-year data collection period, suggesting that from the university’s core business perspective, the IPP is likely to be a valuable avenue for the provision of work integrated learning opportunities for students. Interest in these placement opportunities indicates that the program is sustainable in terms of ensuring that there is sufficient student support for the increasing number of participants. Growth of the IPP requires appropriate levels of staffing to support student learning and the IPP participants.

Access to external funding to deliver and evaluate programs of this nature is challenging. To justify this commitment from the university and to support applications for future funding, evidence is required to demonstrate that the IPP is having the intended impact for participating veterans and their families, and for students. This requires a more structured approach to evaluation than what has previously occurred in the IPP, ensuring the complexities of the program are considered [17]. Within this is the requirement that key stakeholders in the IPP have a shared understanding of the purpose of evaluation and that the evaluation framework is structured to support staff to collect relevant information in a timely manner.

## Limitations

There are a number of limitations to this process evaluation. The most significant limitation was the impact of poor implementation of and adherence to the evaluation framework as originally intended. This resulted in relevant program delivery-specific documentation not always being collected as it should have been between 2017 and 2020, which meant records had to be re-visited and examined to provide the required information. Evaluation issues were exacerbated by staff changes and a lack of clarity around role responsibility. The perspective of the students who undertook placement in the IPP would have been invaluable in describing their experiences and in refining the placement experience. Inclusion of the student perspective is recommended for future evaluation.

## Conclusion

This process evaluation has identified a high level of implementation fidelity and adherence to IPP EaPP-specific activities, a participant and family support network population who were mostly satisfied with the program, and a number of components that were perceived to require amendment or addition. There were key elements of support for veterans–associated with non-selection or return from participation in representative competitions and transition from the EaPP component—that were not provided in early iterations of IPP. Efforts to support the sustainability of IPP will need to be multifaceted and accommodate the needs of veterans and their families while simultaneously meeting the core business of the university: providing educational opportunities for its students. To justify the ongoing expense with respect to the provision of facilities, staff time to manage the program, and staff time to supervise placement students there is a need to demonstrate the effectiveness of the program in supporting veterans to reintegrate and engage in the community, and the impact of the program on the physical and psychological wellbeing of veterans and their families. Concurrently, the program must continue to build capacity to provide placement and work integrated learning opportunities for the university’s students. This process evaluation has demonstrated the need for a more structured approach to the ongoing evaluation of the IPP. This includes ensuring that program staff have a shared understanding of the purpose of evaluation activities and that these activities occur as intended.

## Supporting information

S1 File Interview guide. (PDF)

S2 File Audit trail example. (PDF)

S3 File COREQ checklist. (PDF)
