# Supplementary material for: Process evaluation of the Invictus Pathways Program
Source: PLoS One. 2023 Nov 27;18(11):e0293756. doi: 10.1371/journal.pone.0293756 (PMC10681190; doi:10.1371/journal.pone.0293756)
Supplement: S1 File — (PDF) [file pone.0293756.s001.pdf]

Interview Guide for Invictus Pathways Program Participants

1. Please tell me a little about yourself.
2. Have you been involved in any sports and recreation prior to joining the Invictus Pathways Program? (Prompts: type of sport, length of engagement, frequency, duration, level of achievement/success)
3. What was your life like prior to starting the Invictus Pathways Program?
4. What were your expectations in regards to the Invictus Pathways Program?
5. How did you expect that taking part in the Invictus Pathway Program would affect you? (Prompts: from physical, social and psychological perspective)
6. How do you think that being in the Invictus Pathways Program has impacted your life? (Prompts: from physical, social and psychological perspective)
7. In your opinion, which aspects of the program are most beneficial? Why do you think these aspects are most beneficial?
8. In your opinion, which aspects of the program could be improved? Why do you think these aspects can be improved? How do you think these aspects can be improved?
9. Do you have any further comments you would like to make in relation to what we have talked about?

Interview Guide for Support Network Members

1. Please tell me a little about yourself.
2. Please tell me a little bit about the person you are supporting and what your relationship to him/her is.
3. What was *[insert support person's name]* life like prior to starting the Invictus Pathways Program?
4. What has your relationship with *[insert support person's name]* been like prior to him/her starting the program?
5. What were your expectations in regards to the Invictus Pathways Program?
6. How did you expect that taking part in the Invictus Pathway Program would affect *[insert support person's name]?*
7. How did you expect *[insert support person's name]* participation in the Invictus Pathways Program would affect your relationship with *[insert support person's name]* or you personally?
8. How do you think that being in the Invictus Pathways Program has impacted *[insert support person's name]* life? (*Prompts: from physical, social and psychological perspective*)
9. How do you think that *[insert support person's name*] being in the Invictus Pathways Program has impacted <u>your</u> life and your relationship with *[insert support person's name]*?
10. In your opinion, which aspects of the program are most beneficial? Why do you think these aspects are most beneficial?
11. In your opinion, which aspects of the program could be improved? Why do you think these aspects can be improved? How do you think these aspects can be improved?
12. Do you have any further comments you would like to make in relation to what we have talked about?

Interview guide - Invictus Pathways Program (IPP) staff

1. Can you please tell me a little bit about yourself and your involvement with the IPP (e.g. how
2. long, in what capacity)?
3. From your perspective, what was the IPP’s main objective when it first started?
4. From your perspective, how has the main objective of the IPP changed over time?
5. From your perspective, how has the IPP developed over the last 3 years (e.g. size, services
6. provided, numbers supported, organisational structure)
7. In your opinion, what have been the biggest challenges in relation to the IPP?
8. In your opinion, what are the most beneficial aspects of the IPP?
9. In your opinion, what are the least beneficial aspects of the IPP?
10. Can you please describe any barriers to the IPP’s effectiveness?
11. Can you please describe any potential improvements to the IPP?
12. Do you have any further comments you would like to make in relation to what we have
13. talked about?
