# Supplementary material for: Process evaluation of the Invictus Pathways Program
Source: PLoS One. 2023 Nov 27;18(11):e0293756. doi: 10.1371/journal.pone.0293756 (PMC10681190; doi:10.1371/journal.pone.0293756)
Supplement: S2 File — (PDF) [file pone.0293756.s002.pdf]

Audit trail data analysis example

Example of analysis for the theme: Areas of the IPP requiring improvement and suggestions for change (not all sub-themes included)

Theme	Areas of the IPP requiring improvement and suggestions for change			
Sub-themes	Program communication and scheduling		Person-centred approaches, incorporating mental health awareness	
Categories	Communication within the program	Timing of activities	Difficult for veterans with anxiety to engage/get involved	Lack of understanding of the mental health of veterans
Codes (examples)	Communication is the most important thing		Need to consider participants' anxiety in engaging in offered activities	
	I would love a bit more knowledge about how it works		Some people didn't understand our mental health	
	Timings [of sessions] haven’t been great		Need to be made aware of our triggers	
Quotations	‘Communication is the most important thing. Every time there’s a change of any type, people have to be notified because anytime you get left in the dark and you don’t know what’s going on, you’ll find it more and more frustrating.’		‘…they’re new, they don’t know anybody in the program, they weren’t really connected properly, so for them, especially with some of the anxiety issues and stuff like that that they have, it’s a big thing for them to just show up.’	
	‘The timings [Zoom sessions] haven’t been great because I’ve got other commitments with the kids’ sport and things like that…’		‘…I'm sure they just don’t understand our mental health, and maybe if they're not, maybe they should be made aware of what our triggers are. I definitely felt like at some points in time, I felt like I wasn’t welcome anymore [at service].’	
